# Intraocular lens power calculation for the equine eye

**DOI:** 10.1186/s12917-018-1448-6

**Published:** 2018-04-03

**Authors:** Ulrike Meister, Christiane Görig, Christopher J. Murphy, Hubertus Haan, Bernhard Ohnesorge, Michael H. Boevé

**Affiliations:** 1 Stiftung Tierärztliche Hochschule Hannover, Klinik für Pferde, Bünteweg 9, 30559 Hannover, Germany; 2 De Graafschap dierenartsen, Schimmeldijk 1, 7251 MX Vorden, The Netherlands; 3 UC Davis Health, 2315 Stockton Blvd, Sacramento, CA 95817 USA; 4 HENSOLDT Optronics GmbH, Carl-Zeiss-Str. 22, 73447 Oberkochen, Germany

**Keywords:** Retinoscopy, Keratometry, Ultrasonographic biometry, Intraocular lens, Equine

## Abstract

**Background:**

Phacoemulsification and intraocular lens (IOL) implantation during cataract surgery in horses occur with increasing frequency. To reduce the postoperative refractive error it is necessary to determine the proper IOL power. In the present study retinoscopy, keratometry and ultrasonographic biometry were performed on 98 healthy equine eyes from 49 horses. The refractive state, corneal curvature (keratometry) and the axial location of all optical interfaces (biometry) were measured. The influences of breed, height at the withers, gender and age on values obtained and the comparison between the left and right eye were evaluated statistically. Corresponding IOL power were calculated by use of Binkhorst and Retzlaff theoretical formulas.

**Results:**

Mean ± SD refractive state of the horses was + 0.32 ± 0.66 D. Averaged corneal curvature for Haflinger, Friesian, Pony, Shetland pony and Warmblood were 21.30 ± 0.56 D, 20.02 ± 0.60 D, 22.61 ± 1.76 D, 23.77 ± 0.94 D and 20.76 ± 0.88 D, respectively. The estimated postoperative anterior chamber depth (C) was calculated by the formula C = anterior chamber depth (ACD)/0.73. This formula was determined by a different research group. C and axial length of the globe averaged for Haflinger 9.30 ± 0.54 mm and 39.43 ± 1.26 mm, for Friesian 10.12 ± 0.33 mm and 42.23 ± 1.00 mm, for Pony 8.68 ± 0.78 mm and 38.85 ± 3.13 mm, for Shetland pony 8.71 ± 0.81 mm and 37.21 ± 1.50 mm and for Warmblood 9.39 ± 0.51 mm and 40.65 ± 1.30 mm. IOL power was calculated with the Binkhorst and Retzlaff theoretical formulas. Calculated IOL power for the several breeds ranged from 18.03 D to 19.55 D. The mean value across all horses was 18.73 D determined with Binkhorst formula and 18.54 D determined with Retzlaff formula.

**Conclusions:**

Mean result of this study is: an 18.5 D IOL seemed to be the most appropriate to achieve emmetropia after IOL implantation in horses. Cataract surgery without IOL implantation results in hyperopic and visual compromised horses. Retinoscopy, keratometry and ultrasonographic biometry should be performed on every affected horse and postoperative visual outcome should be determined.

## Background

Cataract has been reported to occur in horses with an estimated incidence of 5% to 7% of all horses with otherwise clinically normal eyes [1]. Cataract can impair visual performance rendering the horse unfit for specific functions, results in devaluation of the individual and can predispose the animal to self-injury [2–4]. Visual function can be restored through surgical extraction of the cataractous lens. Reported methods include aspiration, intracapsular extraction, extracapsular extraction and phacoemulsification with aspiration. Without the implantation of an intraocular lens following cataract extraction horses were left aphakic and were markedly hyperopic [5–7]. Early reports suggested aphakic horses to perform quite well and visual performance to be functionally acceptable [4, 5, 8]. However in other studies significant visual impairment in aphakic horses were detected, which manifested as reduced night vision and reduced contrast sensitivity [3, 9]. In highly hyperopic eyes induced by aphakia, no object in space is accurately imaged on the retina with the degree of defocus worsening as objects are moved closer to the horse’s eye. The mean refractive error in aphakic horses has been reported as approximately + 9.5 D [2–4]. In humans this degree of refractive error corresponds to a Snellen value of 20/ 1200 with 20/200 acuity in the best performing eye of an individual being designated as legally blind [3, 8]. It is reasonable to assume that induction of marked hyperopia associated with lens removal in the horse would dramatically affect visual performance. The degree of defocus scales directly with a decrease in measured visual acuity in humans and dogs [10].

While correction of the refractive error by implantation of an IOL during cataract surgery in humans and dogs is standard therapy, the number of horses, which undergoes phacoemulsification and IOL implantation is much smaller [11–16]. To the author’s knowledge there are currently three different foldable IOLs for horses available. To assist the surgeon in achieving emmetropia in the patient after cataract surgery in humans, presurgical determination of required IOL power is performed with a wide variety of IOLs of differing dioptric power being commercially available. In veterinary patients biometry, keratometry and theoretical formulas have been employed to determine the ideal predicted power of IOL for several species [11, 12, 17–19]. It is important to note that many formulas used to predict required IOL power in human patients are inappropriate for veterinary patients as they incorporate mathematical constants (eg the A constant in the SRK formula) that are not direct applicable to veterinary species. A few studies were done to assess natural variation in the mentioned values in horses with healthy eyes [12, 17, 20, 21].

The purpose of this study was to determine the degree of refraction with retinoscopy, intraocular distances with A-mode (amplitude modulation) ultrasound with horse adjusted ultrasound velocities and corneal curvatures using a video-keratometer in healthy equine eyes. Furthermore the effect of breed, height at the withers, gender, age and differences between the left and right eye was determined. The values were used to calculate the expected IOL power with the Binkhorst and Retzlaff formulas and were compared with results of IOL implantations in reported studies.

## Methods

### Animals and ophthalmologic examination

The study was approved by the ethics commissions of the University of Veterinary Medicine Hannover, Foundation.

98 healthy equine eyes from 49 horses were examined. The horses were private-owned and examined in a darkened stable. Informed consent was obtained from all owners. All horses underwent a dilated (tropicamide 0.5%) ophthalmic examination, including pupillary light reflex, dazzle reflex, menace response, slit-lamp biomicroscopy^1^ and indirect ophthalmoscopy.^2^ Horses with ocular abnormalities were excluded from the study. Only manual restraint was used for all procedures. Following clinical evaluation retinoscopy, keratometry and A-mode ultrasonography were conducted on both eyes of each horse. Prior to the examinations the horses received 1 drop of tropicamide 0.5% ophthalmic solution^3^ and directly in front of the ultrasonographic biometry 1 drop of lidocain ophthalmic solution^4^ was instilled.

### Retinoscopy

Streak retinoscopy was performed with a streak retinoscope^5^ and 2 skiascopy bars.^6^ Streak retinoscopy was performed about 30 min after the administration of tropicamide ophthalmic solution^3^. The horizontal and vertical meridians were refracted at a working distance of 67 cm using the technique described in a previous study [22]. Results of the horizontal and vertical measurements were averaged to calculate a mean refractive error of each eye.

### Keratometry

Keratometry was conducted by using a custom-made video-keratometer.

The video-keratometer consisted of light-emitting diodes (LEDs) placed around a video camera, which recorded the reflected LED image from spherical surfaces. The spherical surfaces were either steel balls, for calibration purpose, or equine eyes producing Purkinje I images. The LEDs emitted in the near infrared, so invisible to the human and equine eye [3]. Switching on and off the LEDs failed to elicit a measurable response of the horse. The video camera was sensible in the near infrared. The light of each LED was projected by a small collimator to the spherical surface, so that the origin of the light appeared to be at infinity. As also mentioned in an earlier study [23] this is mandatory for the virtual image to be located optically at the focus point of the aerial mirror. In this study two horizontal collimators and two vertical collimators were used, so the horizontal and vertical radius of curvature of the equine eye could be measured separately. Each pair of collimators was separated 72 mm and arranged on a circle of 140 mm diameter around a video camera. As the surface acts as an aerial mirror, the distance of two reflected light points in the recording, called image height by opticians, was proportional by similar triangles to the radius of curvature of the sphere. The distance ring of the video camera was locked during all measurements and the apparatus was moved towards the horse eye (or steel ball) until the image was sharp. That was at approximately 300 mm distance from the collimator to the eye (or steel ball). As this method guaranteed always the same distance in repeated measurements, no other distance measurement was necessary, so that the measurement time was minimized for the horse.

By calibration with steel balls all constant factors like the aforementioned distance, the magnification factor of the video camera lens and any magnification of the video software are dumped in a single calibration value and need not be known in detail.

First of all the video-keratometer was calibrated by recording images of light reflected from steel balls of known radii of curvature. In the recordings the distance of the two horizontal and the two vertical reflections were measured in pixels and two (one for the horizontal case and one for the vertical case) calibration curves were generated showing pixel distance over steel ball radius. The calibration curves were linear.

Video-keratometry was performed on both eyes of every horse. The video-keratometer was moved to the horse’s eye until the images were displayed sharp on the monitor of the video camera. The video camera recorded the two horizontal and two vertical light points for a few minutes. After these measurements the recordings were transferred to a computer program for analysis.

The distances of the two horizontal and the two vertical projected light points were measured in the video recordings and served, together with the calibration, to determine the horizontal and vertical radius of curvature and corneal astigmatism of the horse eye.

Three measurements of the radii from each plane (horizontal and vertical) were conducted and averaged. Horizontal corneal curvature (K1) and vertical corneal curvature (K2) was calculated by the eq. K [D] = (N – 1/ radius [m]). The effective corneal refractive index (N) was set at *N* = 1.336 [24]. Averaged corneal curvature (K) was calculated by $\frac{K1+K2}{2}$ in D.

### IOL calculation

The detected data were used to calculate the predicted IOL power [D] by using the Binkhorst theoretical formula and the Retzlaff theoretical formula:

Binkhorst theoretical formula:

$$ Pe=\frac{1336\ \left(4r-L\right)}{\left(L-C\right)\left(4r-C\right)} $$

Pe is the predicted IOL power in D, r is the averaged corneal radius in mm, L is the axial length (AxL) in mm and C is the expected postoperative anterior chamber depth in mm and was calculated by the formula C = ACD/0.73. A previous study [7] reported the mean preoperative-to-postoperative ACD ratio in equine eyes to be 0.73.

Retzlaff theoretical formula:

$$ Pe=\frac{N}{L-C}-\frac{NK}{N- KC} $$

Pe is the predicted IOL power in D, N is the refractive index of aqueous and vitreous (1.336), L is the axial length (AxL) in m, C is the postoperative anterior chamber depth in m and K is the averaged corneal curvature in D.

### A-mode ultrasonography

Measurements of anterior chamber depth (ACD), crystalline lens thickness (LT), vitreous body length (VBL) and axial length (AxL) of the globe was obtained with an ultrasonographic device^7^ with a 10 MHz A-scan probe. Ultrasound velocities were set at 1532 m/s for the anterior chamber and the vitreous body and 1641 m/s for the lens. To achieve more accuracy, values for lens thickness were corrected by the equine-specific conversion factor of 1.008 [25]. Five A-scan recordings of both eyes of each horse were obtained vertically along the optical axis and the values for each eye were averaged. To obtain optimal scans the manual freeze mode was used and the images were frozen when all of the required echoes are present and of sufficient height.

### Statistical analysis

As no significant difference was detected for any measurements between left and right eyes (tested with Wilcoxon-Mann-Whitney-U test), the data from both eyes were averaged for further statistical analyses. Values are reported as mean ± SD. Statistical analyses were made with computerized statistical software.^8^ Normal distribution of data was tested by using the Kolmogorov-Smirnov test. Correlations between age, height at the withers, refractive state, r, ACD, LT, VBL and AxL were evaluated by calculation of Spearman correlation coefficients. The influence of gender and type of horse on refractive state, r and AxL were determined by using the Kruskal-Wallis test. Wilcoxon-Mann-Whitney-U tests were used for comparisons between the horizontal and vertical refractive state and the horizontal and vertical corneal radius in the same eye. Values of *P* ≤ 0.05 were considered significant.

## Results

The examined 49 horses contained 36 mares, 9 geldings and 4 stallions. Ages ranged from 2 to 25 years (mean age = 8.28 ± 5.36 years). Breeds included Haflinger (*n* = 10), Shetland pony (*n* = 7), Warmblood (*n* = 13), Friesian (*n* = 8) and Pony (breed unspecified; *n* = 11). Heights at the withers ranged from 85 to 178 cm (mean height = 145.06 ± 27.26 cm).

### Retinoscopy

Retinoscopy was performed on both eyes of the 49 horses. Results of the horizontal and vertical measurements were averaged. The mean refractive state ±SD of the horses was + 0.32 ± 0.66 D. Emmetropia (− 1 D – 1D) was present in 91 (92.8%) of 98 eyes. Ametropia occurred in 7 (7.2%) eyes, with 4 (4.1%) being myopic with a maximum value of − 5.0 D and 3 (3.1%) being hyperopic with a maximum value of + 1.5 D. Anisometropia (refractive state of the left and right eye differing by more than 0.5 D) occurred in 6 horses (12.2%). In 4 of the anisometropic horses hyperopia was present in one eye, in one horse myopia was present in one eye and in one horse myopia was present in both eyes but of differing magnitudes. There were no significant differences between the horizontal and vertical meridians (*P* = 0.068). No significant influence was found for the breed of horse (*P* = 0.074), gender (*P* = 0.761), height at the withers (*P* = 0.924), age (*P* = 0.917), corneal radius (*P* = 0.201) or globe axial length (*P* = 0.503).

### Keratometry

Horizontal and vertical corneal radii of curvature in the central corneal region were obtained for both eyes in all 49 horses. The appropriate horizontal (K1), vertical (K2) and averaged (K) corneal curvature were calculated and the corneal astigmatism was determined. The mean values ± SD for the different types of horses are shown in Table 1.

**Table 1:** Corneal radius, corneal curvature and corneal astigmatism are depicted depending on breed

Breed	horizontal corneal radius (mm)	vertical corneal radius (mm)	averaged corneal radius (mm)	K1 (D)	K2 (D)	Mean K (D)	corneal astigmatism (D)
Haflinger	16.06 ± 0.47	15.53 ± 0.40	15.79 ± 0.42	20.94 ± 0.62	21.66 ± 0.55	21.30 ± 0.56	0.72 ± 0.36
Friesian	16.94 ± 0.50	16.66 ± 0.49	16.80 ± 0.48	19.86 ± 0.61	20.19 ± 0.62	20.02 ± 0.60	0.34 ± 0.29
Pony	15.16 ± 1.12	14.76 ± 1.34	14.96 ± 1.22	22.29 ± 1.61	22.94 ± 1.95	22.61 ± 1.76	0.65 ± 0.61
Shetland pony	14.46 ± 0.62	13.89 ± 0.57	14.18 ± 0.57	23.30 ± 1.00	24.25 ± 0.97	23.77 ± 0.94	0.95 ± 0.58
Warmblood	16.45 ± 0.66	15.99 ± 0.74	16.22 ± 0.69	20.46 ± 0.84	21.06 ± 0.96	20.76 ± 0.88	0.60 ± 0.35

The horizontal corneal radius of curvature was significantly greater (*P* < 0.001) than the vertical corneal radius indicating the cornea to have an astigmatic curvature, being flatter horizontally than vertically. The differences in horizontal and vertical meridian ranged between 0.34 D and 0.95 D. There was no significant difference between the corneal curvature and the age (*P* = 0.071) of the horses. Corneal curvature did correlate positively, however, with the height of the withers (*P* < 0.001) and the axial length of the globe (*P* < 0.001). A significant influence also existed for the breed of horse (*P* < 0.001) and the gender (*P* = 0.027) on the corneal curvature.

### Ultrasonographic biometry

Biometric values were obtained in both eyes of all horses by use of A-mode ultrasonography. The expected postoperative anterior chamber depth (C) was calculated using the formula $\mathrm{C}=\frac{\mathrm{ACD}}{0.73}$ [7]. Mean values ±SD are provided in Table 2.

**Table 2:** Anterior chamber depth, lens thickness, vitreous body length, axial length and calculated post operative anterior chamber depth are depicted depending on breed

Breed	Anterior Chamber Depth (ACD) (mm)	Lens Thickness LT) (mm)	Vitreous Body Length (VBL) (mm)	Axial Length (AxL) (mm)	C (calculated post operative ACD) (mm)
Haflinger	6.79 ± 0.39	10.91 ± 0.27	21.82 ± 0.94	39.43 ± 1.26	9.30 ± 0.54
Friesian	7.39 ± 0.24	11.70 ± 0.30	23.23 ± 0.97	42.23 ± 1.00	10.12 ± 0.33
Pony	6.34 ± 0.58	11.43 ± 0.82	21.18 ± 2.07	38.85 ± 3.13	8.68 ± 0.78
Shetland pony	6.36 ± 0.58	11.50 ± 0.50	19.45 ± 1.22	37.21 ± 1.50	8.71 ± 0.81
Warmblood	6.85 ± 0.37	11.79 ± 0.50	22.10 ± 0.92	40.65 ± 1.30	9.39 ± 0.51

Significant influences were found between AxL and ACD (*P* < 0.001), LT (*P* < 0.001), VBL (*P* < 0.001), corneal curvature (*P* < 0.001), height at the withers (*P* < 0.001), breed of horse (*P* < 0.001) and age (*P* = 0.008). The larger the AxL the greater the ACD, LT and VBL and the flatter the corneal curvature became and the taller and older the horses were the larger the AxL increased. The gender (*P* = 0.026) also influenced the AxL.

### IOL calculations

For IOL calculation the Binkhorst theoretical formula and the Retzlaff theoretical formula were used. The expected mean IOL dioptric power was calculated using the values for AxL, K and C. The calculated mean IOL dioptric powers for the several breeds of horses are provided in Table 3.

**Table 3:** IOL calculations using Binkhorst and Retzlaff theoretical formulas

Breed	IOL dioptric power derived using Binkhorst formula	IOL dioptric power derived using Retzlaff formula
Haflinger	19.55 D	19.36 D
Friesian	18.20 D	18.03 D
Pony	18.27 D	18.07 D
Shetland pony	19.03 D	18.80 D
Warmblood	18.66 D	18.48 D

## Discussion

Values for corneal curvature, axial length of the globe and postoperative anterior chamber depth of each horse are necessary to calculate the appropriate IOL power with the Binkhorst and Retzlaff theoretical formulas. While streak retinoscopy and ultrasound biometry are relatively simple to perform and routinely used in equine cataract patients, it is rather difficult to determine keratometric data. In order to verify the visual outcome after IOL implantation a post operative evaluation via streak retinoscopy is necessary. Furthermore to establish the exact position of the IOL in the eye and to determine the postoperative anterior champer depth ultrasound measurements after IOL implantation should be performed [2, 26]. Moreover, these values could be used to improve the theoretical formulas with respect to equine cataract patients. In this study retinoscopy, video-keratometry and A-mode ultrasonography with horse adjusted ultrasound velocities were obtained on healthy equine eyes and the corresponding IOL powers were calculated using the Binkhorst and Retzlaff theoretical formulas.

Investigators of 1 study [27] found only changes in the refractive state during the 20 min following the application of topical tropicamid 1%. Hence in the present study streak retinoscopy was performed about 30 min after the application of tropicamide 0.5% ophthalmic solution. As in another study [28] emmetropia was specified as ±1.0 diopter. The mean refractive state ±SD of all horses was + 0.32 ± 0.66 D. In the examined population 92.8% of values for refractive state were within one diopter of emmetropia, 3.1% were hyperopic and 4.1% were myopic. The results are congruous with another recent report [29]. Bracun found that 83.63% were emmetropic, 8.86% were hyperopic and 7.51% were myopic. However in another study [21] emmetropia was present in only 48.7%, hyperopia in 24.1% and myopia in 27.2% of the horses examined, but emmetropia was strictly defined as a refraction of 0 D. In the present study anisometropia (refractive power of the two eyes differs more than 0.5 D) occurred in 6 horses (12.2%).

The values obtained for the averaged corneal curvature (see Table 1) correspond well with one previous report employing photokeratometry [21] but differ by 3.5-8.5 D from two previously reported studies using photokeratometry and B-mode ultrasonography [12, 17]. Differences in corneal curvature values may be influenced by the differing methods employed. Mouney and co-workers documented a significant difference in values obtained by B-mode ultrasonography vs. photokeratometry of approximately 1.2 D [12]. Other differences between the current and previously reported studies include the state of sedation /anesthesia and presence of an auriculopalpebral nerve block [12, 21]. These differences highlight the need to establish standardized instrumentation and methods for performing keratometry.

A significant difference was found between the horizontal and the vertical corneal curvature, which are expected and normal and do not result in differences in the refractive error in the respective meridians. Corneal curvature depended significantly on the breed of horse, the height of the withers and the axial length of the globe. The taller the horse and the larger the axial length of the globe are, the flatter the corneal curvature. The age of the horses had no significant influence on the corneal curvature. In contrast to current findings, Grinniger and co-workers found a positive relationship between age and corneal radius of curvature [21]. A possible reason for the difference between the present study and this previous report is the range of ages included in the studies. In the prior report the group of the youngest horses was between 0 and 2.5 years old while the youngest horse in the present study was 2 years. A similar correlation between age and radius of curvature has been reported for cats. A dramatic decrease in corneal curvature in cats occurs between 9 weeks and 1 year of age [30]. The age of the horse should be considered for IOL selection in very young horses. Townsed et al. [31] found a reduced dioptric strength of the juvenile cornea of horses and a shortened vitreous chamber depth. Hence, to achieve emmetropia in the juvenile eye an IOL with a dioptric strength greater than that required by an adult equine is necessary. However the grown up horses is ametropic. Therefore, it is recommended to use IOL in young horses that will correct vision once the eye reaches its adult parameters.

The present study found a significant correlation between gender and corneal curvature. Such a correlation was not found in a prior study [21]. Results may have been influenced by a much greater number of female (*n* = 36) vs. male (*n* = 13) horses being examined in this study.

A-mode ultrasonography is deemed to be the most accurate method for ocular biometry [32] with B-mode considered inappropriate for ocular biometry [33]. In human cataract surgery, A-mode ultrasound is routinely used in preoperative diagnostic and in postoperative monitoring [34]. Important parameters that can influence the accuracy of A-mode biometry are the values for ultrasound velocity used for determining exact distances. The ultrasound velocity in lens tissue differs considerably in different species. In dogs an underestimation of the lens thickness of 4% results when calculations of lens thickness are made on the basis of ultrasound velocity in the human lens. Use of inaccurate values for velocity can result in incorrect biometric values, which will, in turn, result in incorrect prediction of optimal IOL power to achieve emmetropia [35]. In a previous study [25] species-specific ultrasound velocity for equine aqueous humor, lens and vitreous body were determined. To obtain accurate A-mode biometric values it is recommended to use appropriate values for ultrasound velocity [25]. In the current study, the A-mode instrument utilized ultrasound velocities established for human patients. Hence, the values for lens thickness were corrected by using a conversion factor of 1.008. Intraocular distances (ACD, LT, VBL, AxL) determined in the present study are congruous with prior reports [12, 17, 21, 36].

Cataract surgery with subsequent IOL implantation following phacoemulsification is being performed with increasing frequency in horses [7]. It is a fact that IOL implantation significantly reduces postoperative refractive error [12]. In human medicine several IOL power calculation formulas were developed. They could be classified into theoretical formulas and regression formulas. While the theoretical formulas only were influenced by the variables corneal curvature, axial length, postoperative anterior chamber depth and aqueous and vitreous refractive index, the regression formulas were derived by linear regression analysis of data sets and incorporate mathematical constants [37]. These constants depend on several factors and were not transferable to veterinary species. To the authors` knowledge there are no regression formulas for veterinary medicine available. Hence, the appropriate IOL power for horses was calculated with the Binkhorst and Retzlaff theoretical formulas, despite the fact, that regression formulas show more accuracy than theoretical formulas in human patients [37, 38].

Mean predicted IOL dioptric power for the various types of horses was found to lie between 18.03 D and 19.55 D (see Table 3). Compared to two other studies [12, 17] which calculated an IOL power of 29.91 D (with Binkhorst formula) and 29 D (with Retzlaff formula) and 22 to 23 D by the use of the Binkhorst formula values presented in this study were considerably smaller. Essential factors, which influence the predicted IOL power, are corneal curvature, axial length of the globe and predicted postoperative anterior chamber depth. In the present study, mean corneal curvature ranged over 20.02 – 23.77 D. These values differed from previous reports [12, 17] using different methods for obtaining keratometry results (16.46 D, 16.5 D and 15.3D) by 3.5-8.5 D. Additionally the postoperative anterior chamber depth (C) influences the predicted IOL power. C was calculated by the formula C = ACD/0.73, a value derived from a previous report [7]. In another study Mouney [12] used the measured ACD as the predicted C (6.8 mm). Postoperative ultrasound measurements of the PACD and refractive data obtained via streak retinoscopy are essential to determine actual position of the implanted IOL and to confirm the predictive power of any theoretical calculations. Other investigators [7], who implanted 30- and 25- diopter IOLs, suggested a 18-D IOL is the appropriate power to achieve emmetropia in adult horses. This prediction correlates well with the predicted IOL power in this study. Two previous in vivo studies [15, 16] implanted a 14 D IOL achieve emmetropia within 0.5 D. Currently, there are three foldable IOLs in two powers (+ 14 D and + 18 D) commercially available for horses. Based on current findings and determinations from previous studies [7, 15, 16, 26] the proper IOL power for use in horses lies between + 14 D and + 18.5 D. Additional studies using standardized methods that include preoperative ultrasonographic biometry and keratometry as well as post-operative refraction are necessary to determine the appropriate IOL power and the long-term effects of IOL implantation [7, 15, 16].

## Conclusions

Aphakic horses have been documented to be markedly hyperopic and visually compromised. They have reduced night vision and reduced contrast sensitivity [3, 9]. Soundness is very important for the pleasure and performance horse. A visually compromised horse may show abnormal behavior and the rider faces an unpredictable, frightened animal [39]. Of the commercially available the IOL with 18 D appears to be the most appropriate to achieve emmetropia after IOL implantation in adult horses [7].

## Abbreviations

ACD: Anterior chamber depth
A-mode: Amplitude modulation
AxL: Axial length
B-mode: Brightness modulation
C: Postoperative anterior chamber depth
cm: Centimeter
D: Dioptre
IOL: Intraocular lens
K: Averaged corneal curvature
K1: Horizontal corneal curvature
K2: Vertical corneal curvature
LED: Light-emitting diode
LT: Lens thickness
m: Meter
MHz: Megahertz
mm: Millimeter
n: Number
N: Refractive index (1.336)
Pe: Predicted IOL power
r: Averaged corneal radius
SD: Standard deviation
VBL: Vitreous body length

## Footnotes

1 Kowa-SL 15, Kowa Optimed Deutschland GmbH, Bendemannstr. 9, 40,210 Düsseldorf, Germany

2 Heine Omega 500, Heine Optotechnik, Herrsching, Germany

3 Mydriaticum Stulln, Pharma Stulln GmbH, Werksstr. 3, 92,551 Stulln, Germany

4 Lidocaine, AST Farma B.V., Wilgenweg 7, 3421 TV Oudewater, The Netherlands

5 Heine Beta 200, Heine Optotechnik, Herrsching, Germany

6 Oculus Optikgeräte, Wetzlar, Germany

7 I^3^SYSTEM-ABD, Innovative Imaging Inc., Sacramento, Calif.

8 R Core Team (2016). R: A language and environment for statistical computing. R Foundation for Statistical Computing, Vienna, Austria. URL https://www.R-project.org/
